# Supplementary figures and images for: LncRNA-Associated Genetic Etiologies Are Shared between Type 2 Diabetes and Cancers in the UAE Population
Source: Cancers (Basel). 2022 Jul 7;14(14):3313. doi: 10.3390/cancers14143313 (PMC9313416; doi:10.3390/cancers14143313)

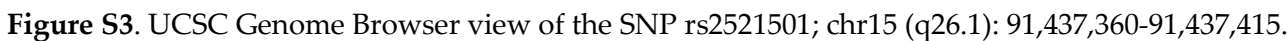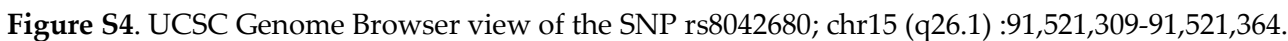

Supplement: Supplementary file 1 [file cancers-14-03313-s001.zip › cancers-1662065-supplementary.pdf]
